# Agriculture: Pesticides Disrupt Nitrogen Fixation

**DOI:** 10.1289/ehp.115-a579a

**Published:** 2007-12

**Authors:** Carol Potera

Talk about a vicious cycle. Some organochlorine pesticides suppress nitrogen-fixing bacteria from replenishing natural nitrogen fertilizer in soil, resulting in lower crop yields, stunted growth, and an ever-greater need for additives to boost production. This previously unrecognized effect stems from pesticides interfering with flavonoid signaling from leguminous plants such as alfalfa, peas, and soybeans to soil bacteria that fix nitrogen. “People assume that endocrine disruption by pesticides occurs only in humans and animals with estrogen receptors, but we find there are nontraditional targets affected by pesticides,” says Jennifer Fox, a postdoctoral researcher at the Center for Ecology and Evolutionary Biology at the University of Oregon.

Plants produce chemicals, many that are structurally similar to phytoestrogens, that attract *Rhizobium* soil bacteria to their root systems to form nodules for nitrogen fixation. Inside the nodules, the bacteria convert atmospheric nitrogen into the natural fertilizer ammonia. These symbiotic soil bacteria respond to plant-produced flavonoid molecules through nodulation D (NodD) receptors, which act similarly to estrogen receptors in vertebrates. In the May 2004 issue of *EHP*, Fox and colleagues reported a study using *Sinorhizobium meliloti*, a soil bacterium that fixes nitrogen in symbiosis with alfalfa. The researchers found that the organochlorine pesticides pentachlorophenol (PCP) and methyl parathion at levels found in farm soils inhibited NodD signaling by 90% and that DDT cut signaling by 45%.

In recent greenhouse experiments, Fox and colleagues at Tulane University treated alfalfa seeds inoculated with *S. meliloti* with PCP, methyl parathion, and DDT, which are found in soil after farmers spray crops, especially in countries where these pesticides have not been banned. Six weeks after treatment, PCP-treated alfalfa plants produced no nodules, and plant yields fell to 17% compared with control plants not treated with pesticides. Methyl parathion and DDT reduced nodule numbers and plant yields by about half. The researchers estimate that the amount of inhibition measured in this experiment could translate in real-world conditions to a one-third loss of plant yield each growing season.

The results, published 12 June 2007 in the *Proceedings of the National Academy of Sciences*, indicate that pesticide residues in soil could not only reduce harvest yields, but also increase the need for synthetic fertilizers, thereby raising costs for farmers and contributing to environmental pollution. The observations also may explain a trend in the past 40 years toward stagnant crop yields despite record high use of pesticides and synthetic fertilizers worldwide.

In unpublished experiments, co-author John McLachlan, director of the Tulane Center for Bioenvironmental Research, showed that the same three pesticides disrupt NodD signaling in *Rhizobium* sp. strain NGR234, a bacterium that fixes nitrogen in symbiosis with more than 100 leguminous plants growing in tropical and subtropical soils. “Many of the species that NGR234 nodulates are trees and bushes, such as teak and rosewood, that promote vital soil improvement within nutrient-poor tropical soils,” he says. McLachlan says farmers in poorer countries especially cannot afford to lose natural fertilizer because synthethic fertilizers cost so much.

The connection between pesticides and nitrogen fixation shows that “pristine and natural interactions between bacteria and plants are being jeopardized by what we put into the soil,” says Ann Hirsch, a plant molecular biologist at the University of California, Los Angeles. Similarly, the effects of pesticides on human disease continue to be documented, with a report in the October 2007 issue of *EHP* linking childhood exposure to DDT with a fivefold higher risk of breast cancer in women. “Now we are affecting our agricultural assets,” Hirsch says, “something people take for granted.”

Fox plans to test the effects of pesticides on alfalfa and soybeans under real-life field conditions. “We want to screen pesticides to see which ones cause minimal damage to certain types of crops and maximize the amount of natural fertilizer made,” she says.

## Figures and Tables

**Figure:**
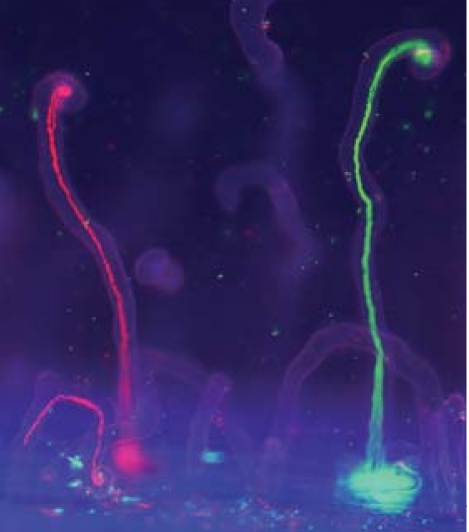
*Rhizobium* infects alfalfa roots

